# 

**Published:** 1908-01-04

**Authors:** 


### CONTENTS.

| | PAGE |
|---|---|
| **Bacteriology and the Medical Treatment of Disease** | 355 |
| **Quackery and its Prevention** | 356 |
| **Annotations—** Certification of Inebriates—Out Patient Departments and the Poor Law—Pharmacology and Therapeutics of the Formates | 357 |
| **Medical Opinion and Movement** | 358 |
| **Hospital Clinics—** Sleeping Sickness. By George C. Low, M.A., M.B. | 359 |
| **Points in Pathology—** Blood Coagulation time in Disease | 363 |
| **Points in Treatment—** Two Simple Methods of Curing Pediculosis Capitis Quickly | 364 |
| **Points in Surgery—** Empyema—II. | 365 |
| Some Diseases of the Male Genital System | 366 |
| **Gynæcology—** Retroversion of the Gravid Uterus | 367 |
| **Dermatology—** The General Examination of the Child's Skin | 368 |
| **Laryngology and Rhinology—** Rhinitis Sicca | 369 |
| **Notes on Current Neurology—** Rudimentary Forms of Disseminated Sclerosis | 370 |
| **Therapeutics—** Secret Remedies and Branded Products | 371 |
| **Resident Medical Officers' Department—** The R.M.O. and the County Court | 372 |
| **Hospital Administration—** Hospital Progress in 1907 | 373 |
| Hospital Construction | 374 |
| News and Coming Events | 377 |
| Social and Poor-law Problems | 377 |
| **Nursing Administration—** Self-Sacrifice or Cowardice | 378 |
| **Editor's Letter-Box—** "Soot and Smoke" | 379 |

